# Supplementary material for: Genome-scale metabolic modeling of Aspergillus fumigatus strains reveals growth dependencies on the lung microbiome
Source: Nat Commun. 2023 Jul 20;14:4369. doi: 10.1038/s41467-023-39982-5 (PMC10359302; doi:10.1038/s41467-023-39982-5)
Supplement: Supplementary file 1 — Supplementary Information [file 41467_2023_39982_MOESM1_ESM.pdf]

Supplementary Information

Supplementary Figures

Supplementary Figure S1

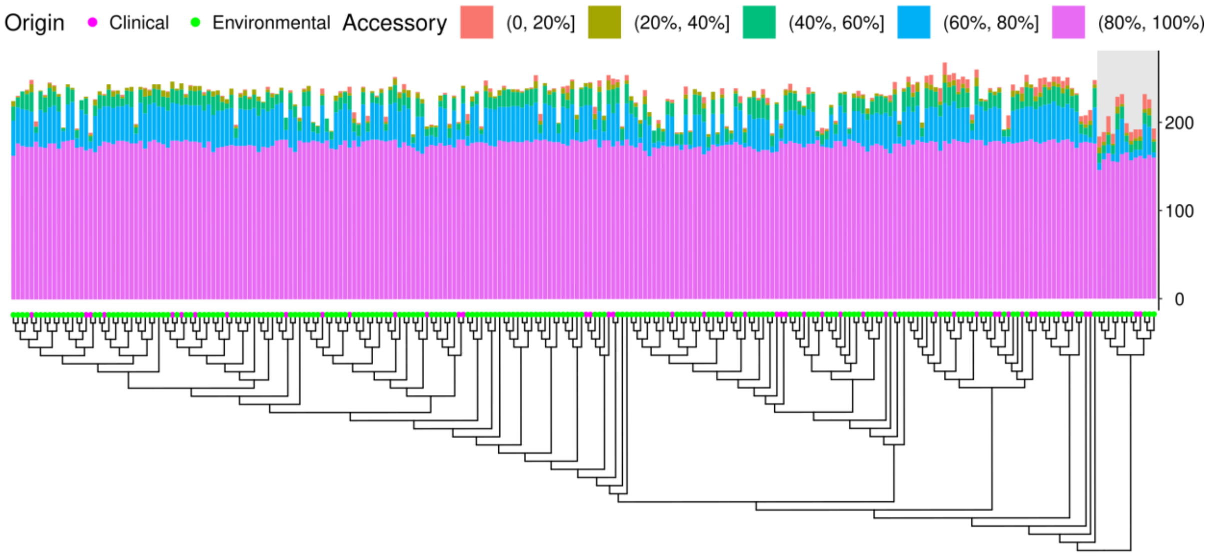

Accessory genome conservation among 252 *A. fumigatus* strains. Indicated percentage ranges denote accessory gene presence across the genomes of all strain-specific GEMs. Cluster with gray background denotes genetic lineage of *A. fumigatus* with significantly fewer accessory genes than other lineages as previously published[1]. Source data for Figure S1 are provided as a Source Data file.

Supplementary Figure S2

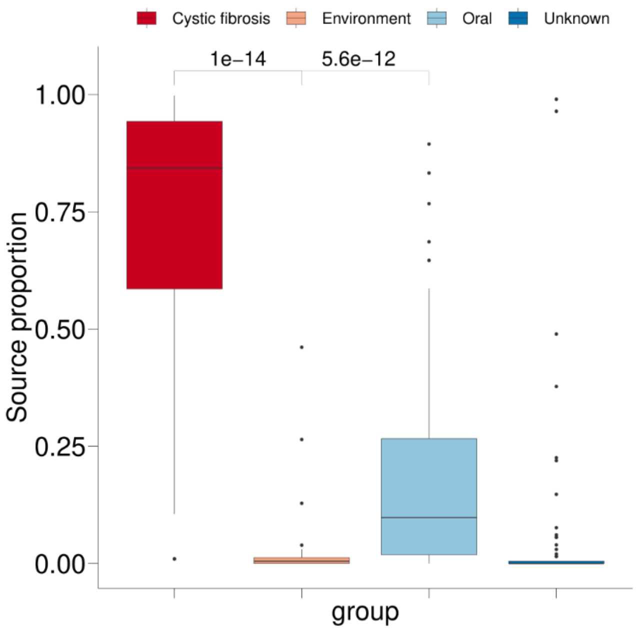

FEAST result for estimating the contribution of potential source environments by combining three published cystic fibrosis lung microbiome datasets and a clinical environmental dataset (see Methods). The group “Cystic fibrosis” (n=85) is the contribution of the cystic fibrosis lung microbiome for each sample, and the group “Environment” (n=24) is the contribution of the clinical environment for each sample. "Oral" (n=132) is the contribution of the oral microbiome for each sample. The group “unknown” is the contribution of other environments of unknown origin. The p value was obtained by two-tailed Wilcoxon matched-pairs signed-rank test. Box-plot elements: center line: median, lower/upper bound: $25^{th}/75^{th}$ percentile, whiskers: minimum and maximum values within 1.5 × interquartile range (IQR), outliers: points outside ±1.5 × IQR. Source data for Figure S2 are provided in the Source Data file.

Supplementary Figure S3

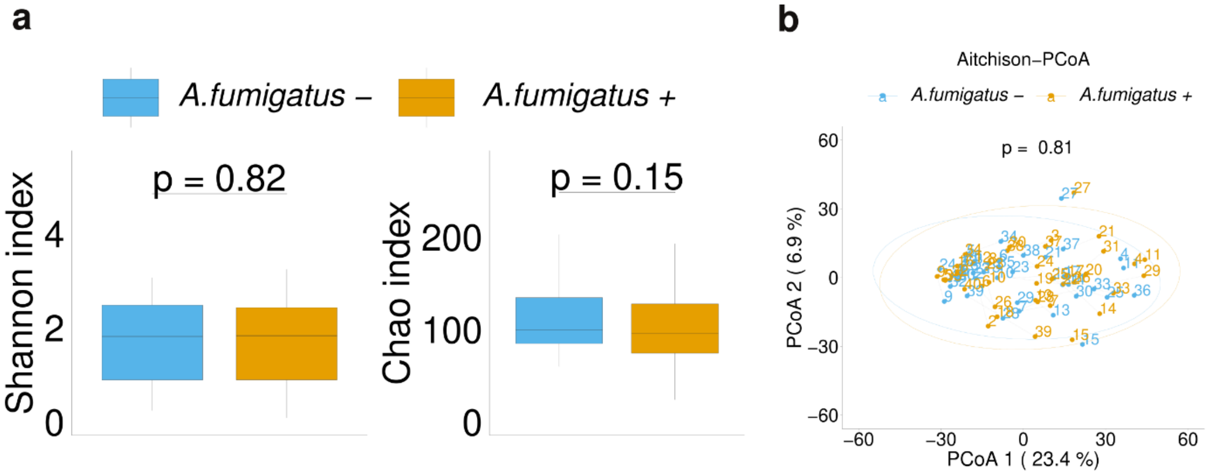

Alpha and beta diversity of cystic fibrosis lung microbiome. a, Statistical significance according to Shannon and Chao diversity index for alpha diversity (n=40 for samples without (-) and with (+) *A. fumigatus* infection). **b**, Beta diversity (Aitchison distance) based on Kraken-derived taxonomic profiles. Two-tailed Wilcoxon signed-rank test was used for alpha diversity comparisons; PERMANOVA was used to assess the statistical significance of beta diversity comparisons. Box-plot elements: center line: median, lower/upper bound: $25^{th}/75^{th}$ percentile, whiskers: minimum and maximum values within 1.5 × interquartile range. Source data for Figure S3 are provided in the Source Data file.

References

1. Barber, A. E. *et al.* Aspergillus fumigatus pan-genome analysis identifies genetic variants associated with human infection. *Nature Microbiology 2021 6:12* **6**, 1526–1536 (2021).
