# Supplementary material for: Genome-scale metabolic modeling of Aspergillus fumigatus strains reveals growth dependencies on the lung microbiome
Source: Nat Commun. 2023 Jul 20;14:4369. doi: 10.1038/s41467-023-39982-5 (PMC10359302; doi:10.1038/s41467-023-39982-5)
Supplement: Supplementary file 3 — Description of Additional Supplementary Data Files [file 41467_2023_39982_MOESM3_ESM.pdf]

Description of Additional Supplementary Data Files

File name: Supplementary Data S1
Description: Biomass composition

File name: Supplementary Data S2
Description: Details on phenotypic growth assays, gene essentiality data and quantitative predictability

File name: Supplementary Data S3
Description: *A. fumigatus* strain metadata

File name: Supplementary Data S4
Description: Details on experimental data including metabolomics, radial growth and metabolic activity

File name: Supplementary Data S5
Description: Machine learning model using minimal media

File name: Supplementary Data S6
Description: Metagenomics of cystic fibrosis samples

File name: Supplementary Data S7
Description: Metadata of clinical cystic fibrosis samples

File name: Supplementary Data S8
Description: Metabolite analysis using MAMBO

File name: Supplementary Data S9
Description: Detailed GEM information

File name: Supplementary Data S10
Description: Constraint details corresponding to specific *in silico* simulations
