# Supplementary material for: Genome-scale metabolic modeling of Aspergillus fumigatus strains reveals growth dependencies on the lung microbiome
Source: Nat Commun. 2023 Jul 20;14:4369. doi: 10.1038/s41467-023-39982-5 (PMC10359302; doi:10.1038/s41467-023-39982-5)
Supplement: Supplementary file 14 — Reporting Summary [file 41467_2023_39982_MOESM14_ESM.pdf]

nature portfolio

Corresponding author(s): Gianni Panagiotou <Gianni.Panagiotou@leibniz-hki.de> Sascha Schäuble <Sascha.Schaeuble@leibniz-hki.de>

Last updated by author(s): Jun 23, 2023

Reporting Summary

Nature Portfolio wishes to improve the reproducibility of the work that we publish. This form provides structure for consistency and transparency in reporting. For further information on Nature Portfolio policies, see our Editorial Policies and the Editorial Policy Checklist.

Statistics

For all statistical analyses, confirm that the following items are present in the figure legend, table legend, main text, or Methods section.

n/a	Confirmed	
☐	☒	The exact sample size (*n*) for each experimental group/condition, given as a discrete number and unit of measurement
☐	☒	A statement on whether measurements were taken from distinct samples or whether the same sample was measured repeatedly
☐	☒	The statistical test(s) used AND whether they are one- or two-sided<br>*Only common tests should be described solely by name; describe more complex techniques in the Methods section.*
☐	☒	A description of all covariates tested
☐	☒	A description of any assumptions or corrections, such as tests of normality and adjustment for multiple comparisons
☐	☒	A full description of the statistical parameters including central tendency (e.g. means) or other basic estimates (e.g. regression coefficient) AND variation (e.g. standard deviation) or associated estimates of uncertainty (e.g. confidence intervals)
☐	☒	For null hypothesis testing, the test statistic (e.g. *F*, *t*, *r*) with confidence intervals, effect sizes, degrees of freedom and *P* value noted<br>*Give P values as exact values whenever suitable.*
☒	☐	For Bayesian analysis, information on the choice of priors and Markov chain Monte Carlo settings
☐	☒	For hierarchical and complex designs, identification of the appropriate level for tests and full reporting of outcomes
☒	☐	Estimates of effect sizes (e.g. Cohen's *d*, Pearson's *r*), indicating how they were calculated

Our web collection on statistics for biologists contains articles on many of the points above.

Software and code

Policy information about availability of computer code

Data collection	No specific software was used to collect data.
Data analysis	The source code for all data analysis can be found in the following repositories: https://zenodo.org/record/8034128, https://github.com/mohammadmirhakkak/A_fumigatus_GEM/.

For manuscripts utilizing custom algorithms or software that are central to the research but not yet described in published literature, software must be made available to editors and reviewers. We strongly encourage code deposition in a community repository (e.g. GitHub). See the Nature Portfolio guidelines for submitting code & software for further information.

Data

Policy information about availability of data

All manuscripts must include a data availability statement. This statement should provide the following information, where applicable:

- Accession codes, unique identifiers, or web links for publicly available datasets
- A description of any restrictions on data availability
- For clinical datasets or third party data, please ensure that the statement adheres to our policy

The authors declare that the data supporting the findings of this study are available within the paper and its Supplementary Information files. Information on metabolic models generated in this study are provided in Supplementary Data S1 and S9. All metabolic models are available at the BioModels repository (ID MODEL2211100001, https://www.ebi.ac.uk/biomodels/MODEL2211100001). Information on phenotypic growth assays generated in this study are provided in

Supplementary Data S2. Information on experimental data including metabolomics, radial growth and metabolic activity generated in this study are provided in Supplementary Data S4. Information on metagenomics of cystic fibrosis samples generated in this study are provided in Supplementary Data S6 and S7. All shotgun metagenomics of sputum from 40 cystic fibrosis patients are available at the European Nucleotide Archive (project ID PRJEB54014, https://www.ebi.ac.uk/ena/browser/view/PRJEB54014). External microbiome datasets analyzed in this study were retrieved from the European Nucleotide Archive (project URLs: https://www.ebi.ac.uk/ena/browser/view/PRJEB38221, https://www.ebi.ac.uk/ena/browser/view/PRJNA316588, https://www.ebi.ac.uk/ena/browser/view/PRJEB32062, https://www.ebi.ac.uk/ena/browser/view/PRJEB28422, https://www.ebi.ac.uk/ena/browser/view/PRJNA376580). Source data are provided with this paper.

Human research participants

Policy information about studies involving human research participants and Sex and Gender in Research.

Reporting on sex and gender	Our findings on cystic fibrosis apply to both sexes as samples from both were included in our study (15 females and 25 males). Sex was assigned based on self-reporting. Sex and further metadata are included in Supplementary Table S7.
Population characteristics	All samples considered were from patients diagnosed with cystic fibrosis. The age characteristics of our patients at the time of sample collection was mean = 23.59 years (standard deviation [SD] = 4.95) before A. fumigatus infection and mean = 24.16 years (SD = 5.12) after A. fumigatus infection. BMI was mean = 20.16 (SD = 2.82) before and mean = 19.99 (SD = 2.57) after A. fumigatus infection.<br>All metadata per patient are available in the supplement of this manuscript (Supplementary Table S7).
Recruitment	Patients were treated according to the standard of care. The diagnosis of cystic fibrosis was verified by established diagnostic criteria. Spontaneously expectorated sputum was collected during visits to the Cystic Fibrosis Center at the University Hospital Heidelberg and frozen in liquid nitrogen on the day of visit. Sputum samples were collected from 40 cystic fibrosis patients before and after they had positive A. fumigatus colonization. Samples were frozen within 24 hr after reception at the microbiology department. The cohort for 80 total samples was 15 females and 25 males aged 23.6 ± 4.96 years (mean ± standard deviation) before A. fumigatus colonization. Samples were taken during visits from patients without exacerbation or intravenous antibiotic treatment in the previous 3 months.
Ethics oversight	This study was approved by the Ethics Committee of the University of Heidelberg and written informed consent was obtained from all patients or their parents or legal guardians (S 370/2011).

Note that full information on the approval of the study protocol must also be provided in the manuscript.

Field-specific reporting

Please select the one below that is the best fit for your research. If you are not sure, read the appropriate sections before making your selection.

[x] Life sciences　[ ] Behavioural & social sciences　[ ] Ecological, evolutionary & environmental sciences

For a reference copy of the document with all sections, see nature.com/documents/nr-reporting-summary-flat.pdf

Life sciences study design

All studies must disclose on these points even when the disclosure is negative.

Sample size	Our study design for cystic fibrosis patients was unique in obtaining samples from patients before and after they had A. fumigatus infection yielding a set of paired samples. Given the pilot nature of our study, no sample size calculation was performed for lung microbiome samples before and after A. fumigatus infection of cystic fibrosis patients. However, all available samples (40 patients, 2x40 = 80 samples for before and after A. fumigatus infection) were considered to allow for confidence in assignment of lung microbial species and maximum statistical power with a paired test design.<br>Similarly or lower sized studies of the lung microbiome were used before and compared against in this study (Pust et al., 2020, doi.org/10.1038/s41522-020-00171-7, 52 samples; Feigelman et al., 2017, doi.org/10.1186/s40168-017-0234-1, 17 samples; Dmitrijeva et al., 2021, doi.org/10.1128/mBio.02863-20, 25 samples of 4 subjects).
Data exclusions	No data were excluded.
Replication	All in vitro findings were based on 7-12 biological replicates. All replication attempts were successful.<br>Taxonomy assignments of the lung microbiomes were cross-checked and confirmed with two alternative tools.
Randomization	Randomization was not necessary as grouping of cystic fibrosis samples was based on detection of A. fumigatus in culture.
Blinding	Blinding was not necessary as grouping of cystic fibrosis samples was based on detection of A. fumigatus in culture.

Reporting for specific materials, systems and methods

We require information from authors about some types of materials, experimental systems and methods used in many studies. Here, indicate whether each material, system or method listed is relevant to your study. If you are not sure if a list item applies to your research, read the appropriate section before selecting a response.

Materials & experimental systems

n/a	Involved in the study
☒	☐ Antibodies
☒	☐ Eukaryotic cell lines
☒	☐ Palaeontology and archaeology
☒	☐ Animals and other organisms
☒	☐ Clinical data
☒	☐ Dual use research of concern

Methods

n/a	Involved in the study
☒	☐ ChIP-seq
☒	☐ Flow cytometry
☒	☐ MRI-based neuroimaging
